# airpg: automatically accessing the inverted repeats of archived plastid genomes

**DOI:** 10.1186/s12859-021-04309-y

**Published:** 2021-08-21

**Authors:** Tilman Mehl, Michael Gruenstaeudl

**Affiliations:** 1 Institut für Bioinformatik, Freie Universität Berlin, 14195 Berlin, Germany; 2 Institut für Biologie, Freie Universität Berlin, 14195 Berlin, Germany

**Keywords:** Data mining, Inverted repeats, NCBI Nucleotide, Plastid genomes, Sequence annotations

## Abstract

**Background:**

In most flowering plants, the plastid genome exhibits a quadripartite genome structure, comprising a large and a small single copy as well as two inverted repeat regions. Thousands of plastid genomes have been sequenced and submitted to public sequence repositories in recent years. The quality of sequence annotations in many of these submissions is known to be problematic, especially regarding annotations that specify the length and location of the inverted repeats: such annotations are either missing or portray the length or location of the repeats incorrectly. However, many biological investigations employ publicly available plastid genomes at face value and implicitly assume the correctness of their sequence annotations.

**Results:**

We introduce airpg, a Python package that automatically assesses the frequency of incomplete or incorrect annotations of the inverted repeats among publicly available plastid genomes. Specifically, the tool automatically retrieves plastid genomes from NCBI Nucleotide under variable search parameters, surveys them for length and location specifications of inverted repeats, and confirms any inverted repeat annotations through self-comparisons of the genome sequences. The package also includes functionality for automatic identification and removal of duplicate genome records and accounts for taxa that genuinely lack inverted repeats. A survey of the presence of inverted repeat annotations among all plastid genomes of flowering plants submitted to NCBI Nucleotide until the end of 2020 using airpg, followed by a statistical analysis of potential associations with record metadata, highlights that release year and publication status of the genome records have a significant effect on the frequency of complete and equal-length inverted repeat annotations.

**Conclusion:**

The number of plastid genomes on NCBI Nucleotide has increased dramatically in recent years, and many more genomes will likely be submitted over the next decade. airpg enables researchers to automatically access and evaluate the inverted repeats of these plastid genomes as well as their sequence annotations and, thus, contributes to increasing the reliability of publicly available plastid genomes. The software is freely available via the Python package index at http://pypi.python.org/pypi/airpg.

**Supplementary Information:**

The online version contains supplementary material available at 10.1186/s12859-021-04309-y.

## Background

The plastid genome is one of three genomes common to all plant cells. In flowering plants, the structure of the plastid genome is relatively conserved and characterized by a duplication and reverse-complemental re-insertion of a section that is approximately 15–25 kb in length, resulting in an unequal quadripartite genome structure [1]. Thus, the typical plastid genome of flowering plants comprises a large (LSC) and a small single copy (SSC) region, separated by two identical inverted repeats (IRs) [2]. The two copies of the IR per plastid genome (i.e., ${\text{IR}}_{\mathrm{A}}$ and ${\text{IR}}_{\mathrm{B}}$) have been found to display identical length and sequence per genome across most lineages of flowering plants [1] and evolutionary time [3]. Of the approximately 120–130 genes encoded in the plastid genome of most photoautotrophic land plants, 9–19 are typically located in the IRs and, thus, duplicated [4]. Equality in length and sequence between the IRs of a plastid genome is likely the result of a frequent sequence homogenization via recombination-dependent replication or related forms of repeat-mediated recombination that may act both within and across plastome molecules [5]. Every plastid organelle comprises multiple copies of the plastid genome as well as a DNA repair and recombination machinery that contains the enzymatic means and ample template to restore the original sequence if mutations were to occur [6]. Several studies have suggested that both gene conversion and copy correction mechanisms continuously operate on the IRs [7]. On the level of genes, such a sequence homogenization exhibits the effect of rapid gene conversion if mutations are artificially introduced [8] and has also contributed to the maintenance of small inversions and secondary DNA structures across evolutionary time [9]. The differential rate of nucleotide substitution between the single copy (SC) and the IR regions of the plastid genome is likely another manifestation of this homogenization process [1]. With a few exceptions [10], any observation of non-identity among the two copies of the IR is, thus, more likely the result of a sequencing or annotation error than a sudden lapse in sequence homogenization. The plastid genome presented by Dempewolf et al. [11], for example, exhibits nucleotide polymorphisms between the un-annotated IRs and represents one of the many cases where plastid genomes with either non-identical IRs or incomplete IR annotations were submitted to public sequence databases without highlighting the observed differences [12]. The expectation of IR equality in complete and correct plastid genomes is also manifest in various software tools for plastome visualization. The software OGDraw [13], for example, employs exact string-matching when determining the location of the IRs within the genome during the plotting of complete plastid genomes and dismisses sequence regions that contain nucleotide polymorphisms from consideration as possible IRs. Similarly, the software Chloroplot [12] operates under the assumption of IR equality in plastid genomes and explicitly highlights the differences between IRs that are found to be non-identical. By the same logic, equality in length and sequence between the IRs of a plastid genome can be used as a measure of sequence and assembly quality of the genome [14]. Knowledge of the exact length and location of the IRs in a plastid genome is also necessary to explore the biological significance of the IR regions and, by extension, the genetic and evolutionary mechanisms maintaining the quadripartite structure of these genomes. Consequently, plastid genomes stored on public sequence repositories should contain complete and correct annotations regarding IR length and location [15].

The comparative analysis of plastid genomes has become a common tool in plant research and has fostered the sequencing of thousands of such genomes in recent years. Contemporary plastid genomic studies are sequencing and analyzing hundreds, if not thousands, of complete plastid genomes per investigation [16–18]. The number of plastid genomes that have been submitted to, and are available from, public sequence repositories such as NCBI Nucleotide (https://www.ncbi.nlm.nih.gov/nucleotide/) has, thus, increased dramatically over the past years [19]. By the end of 2020, there were 14,716 complete plastid genomes of flowering plants stored on NCBI Nucleotide, of which 9483 were unique genome records. This large collection of plastid genomes opens the door for exploring fundamental questions in plant evolution and represents a valuable genomic resource [20]. However, the annotation information deposited and stored alongside many of these genome sequences is not complete and sometimes even inaccurate [19]. Several previous investigations have reported the observation of incorrect annotations among publicly available plastid genomes [21–23]. Hence, it stands to reason that incomplete or incorrect annotations may be more than an occasional occurrence among plastid genomes on NCBI Nucleotide [19]. The exact number of published plastid genomes that exhibit erroneous sequence annotations is difficult to quantify and may be similar to those of erroneous mammalian mitochondrial genomes [24]. It is imperative to identify incomplete or incorrect plastid genome records as early as possible so that other investigations do not include them in their analyses and, thus, invalidate their conclusions [25]. The correct annotation of structural features of plastid genomes (such as the IRs) represents a important aspect in generating accurate and verified genome sequence collections [15].

Surveying the accuracy of sequence annotations of plastid genomes on NCBI Nucleotide can only be achieved through the application of analysis strategies based on the concepts of genome data mining [26]. Specifically, bioinformatic workflows need to be applied that are efficient enough to process large quantities of genome data and flexible enough to accommodate the idiosyncrasies of plastid genome structure. This investigation presents the development and application of such workflows: we design a software tool that contains the necessary functions to automatically access the thousands of plastid genomes stored on NCBI Nucleotide and conduct data mining on their nucleotide records, sequence annotations, and sequence metadata to assess the complete- and correctness of their IR annotations. Specifically, we develop a Python package, titled airpg (short for ‘automatically accessing the inverted repeats of archived plastid genomes’), that surveys the IR annotations of all plastid genomes stored on NCBI Nucleotide in an automated fashion. The package can retrieve complete plastid genomes stored on the database using a flexible search interface, survey the retrieved genomes for sequence annotations of the IRs (or, in their absence, the SC regions), parse the identified SC/IR junction sites as well as IR length and location information, confirm any IR annotations through sequence self-comparisons, and tabulate the identified IR locations (if any) for subsequent statistical analysis. To illustrate the features of airpg and their applicability on empirical data, we conduct a survey of the IR annotations of all complete and verified plastid genomes of flowering plants submitted to NCBI Nucleotide between the beginning of 2000 and the end of 2020 with the aim of identifying possible correlations between incomplete IR annotations and external factors such as release year, publication status, and version number of the genome records.

## Implementation

### IR identification

airpg contains functionality to identify and parse the IR annotations of plastid genomes despite the use of different annotation formats and conventions by different researchers over time. At the core of airpg is the automated identification of the IR regions of a plastid genome record using a multitude of identification criteria based on different annotation features. The application of multiple identification criteria is necessitated by the different annotation formats and conventions that researchers have employed over time when submitting plastid genomes to NCBI Nucleotide. For example, considerable differences in the IR annotations exist among the 14 plastid genome records of wheat (*Triticum aestivum* L.) available on NCBI Nucleotide: an older record (AB042240, submitted in 2000) contains annotations for each of the four junctions between the SC and the IR regions but not for any of these regions themselves, whereas a more recent record (MH051715, 2018) contains annotations for the junction sites as well as for ${\text{IR}}_{\mathrm{B}}$ but not for ${\text{IR}}_{\mathrm{A}}$. A similar situation exists in the IR annotations of the 19 plastid genome records of potato (*Solanum tuberosum* L.) available on NCBI Nucleotide: the feature key *repeat_region* is employed to specify the IR regions in several recent submissions (MT511702–MT511710, all submitted in 2020) but older submissions (e.g., DQ231562, 2006; DQ386163, 2009) use the generic feature key *misc_feature* to annotate the IR and the SC regions, even though that key is a catch-all feature tag intended for features without a dedicated feature key. Evidently, the annotation of IRs in plastid genomes is not fully standardized on NCBI Nucleotide, and, thus, a multitude of different criteria are required to identify and parse IR annotations across a large number of sequence records.

The criteria employed by airpg to identify the IR regions of a plastid genome record can be summarized into one explicit and two implicit processes. In the explicit process, airpg infers the location of the IRs by searching for the annotation feature keys *repeat_region* and *misc_feature*, which must contain the feature qualifier *note* and the qualifier values ‘*inverted repeat A*’ or ‘*inverted repeat B*’ (or their abbreviations ‘*IRa*’ or ‘*IRb*’, respectively). In the implicit processes, airpg uses the annotations of other regions within a quadripartite genome as a corollary for identifying the location of the IRs. Specifically, the implicit processes identify the IR location via either the locations of the LSC and the SSC, or the four junctions flanking these two regions. If the start and end locations of the SC regions are known, the start and end locations of the IRs can be inferred automatically: the end of the LSC denotes the start of the ${\text{IR}}_{B}$, whereas the start of the LSC denotes the end of the ${\text{IR}}_{A}$. Likewise, the end of the SSC denotes the start of the ${\text{IR}}_{A}$, whereas the start of the SSC denotes the end of the ${\text{IR}}_{B}$. Similarly, the start of the LSC is also the start of the entire sequence, whereas the end of the ${\text{IR}}_{A}$ is also end of the entire sequence. In the first implicit process, airpg aims to identify the SC regions directly by searching for any annotation feature that contains the feature qualifier *note* as well as the qualifier values ‘*short single copy*’ or ‘*large single copy*’ (or their abbreviations ‘*ssc*’ or ‘*lsc*’, respectively). In the second implicit process, airpg aims to identify the SC regions indirectly by searching for any annotation feature that contains the feature qualifier *note* as well as qualifier values that denote junction sites via specific junction identifiers. Two types of junction identifiers are scanned for: hard identifiers which comprise verbatim junction abbreviations or the exact names of the flanking regions (e.g., ‘jlb’, ‘lsc-irb’, and ‘irb-lsc’ for the junction between the LSC and ${\text{IR}}_{B}$), and soft identifiers which comprise the general names of flanking regions (e.g., ‘lsc-ir’ and ‘ir-lsc’ for any junction involving the the LSC) and are followed by an inference of their precise location based on their nucleotide position in the genome (with the repeat feature with the numerically lower start position automatically defined as ${\text{IR}}_{B}$). Differences in the capitalization of any keywords employed in the IR identification processes are automatically compensated for by the software. The workflow governing the order that the individual identification processes are executed in is vizualized in Additional file 1: Figure S1.

### Duplicate removal

airpg contains functionality to automatically identify and remove duplicate plastid genomes records during a survey. Among the thousands of sequence records that a scientist retrieves when searching NCBI Nucleotide for complete genome sequences is a substantial number of duplicate records. These duplicates are the result of the NCBI Reference Sequence (‘RefSeq’) database, which is probed during standard searches of NCBI Nucleotide. The NCBI RefSeq database contains a non-redundant set of sequences of chromosomes, complete genomes, and genomic contigs, among others, that serve as reference standards for other sequencing projects [27]. Hence, a query for complete plastid genomes on NCBI Nucleotide will often return the original record of each genome in addition to its RefSeq record. For example, a standard search on NCBI Nucleotide for all plastid genomes of flowering plants published in 2019 returns a total of 3,495 records, of which 1168 (33%) represent duplicate records. To identify and remove duplicate records during automated searches of complete plastid genomes, airpg harnesses the information of the flatfile field ‘COMMENT’ of RefSeq records, which specifies the accession number of the sequence it is referencing. To avoid counting both records, airpg parses the original accession number from the field ‘COMMENT’ and appends it to a list of duplicates for subsequent removal from the search results. By comparison, a survey of the curated database NCBI Genome (https://www.ncbi.nlm.nih.gov/genome/browse#!/organelles/) would not be an alternative solution to the problem of duplicate genome records: NCBI Genome contains only a subset of the plastid genomes that are stored on NCBI RefSeq and, by extension, are accessible via NCBI Nucleotide. Probing NCBI Genome instead of NCBI Nucleotide would, thus, not allow a comprehensive survey of all plastid genomes of flowering plants stored on NCBI Nucleotide (Additional file 2).

### Blocklisting taxa

airpg contains functionality to automatically identify and remove sequence records of taxa that genuinely do not contain IRs in their plastid genomes. While the great majority of flowering plants exhibit plastid genomes with IRs, there are a number of species that lack them naturally [1]. Prominent examples are the members of the ‘inverted repeat-lacking’ (IRL) clade of the Fabaceae [28] as well as taxa in other plant families [29–32]. The plastid genomes of such taxa must be excluded from a survey with airpg, as the algorithm would identify these genomes as insufficiently annotated, even though their lack of IR annotation is the result of a genuine absence of IRs. To avoid the incorrect determination of missing annotations, airpg includes functionality to exclude taxa from a survey based on a user-defined taxon list. Specifically, the package accepts a user-submitted blocklist of species and genus names that must be excluded from any search results. A basic blocklist of taxa that naturally lack IRs is co-supplied with the package; to generate this list anew or extend it, airpg enables users to automatically query NCBI PubMed for publications that describe IR loss among plant taxa and, thus, add additional IRL taxa to the blocklist. Specifically, the software can evaluate the titles of PubMed publications for keywords (such as ‘IR’, ‘inverted repeat’, ‘lack’, ‘loss’, and any combination thereof) to identify publication abstracts that contain the names of plant genera that naturally lack IRs. Once such abstracts are identified, they are parsed for genuine plant names by comparing each proper name against a local copy of the NCBI taxonomy database. Species and genus names can also be added manually to the blocklist at any time. In the event that both a plant genus as well as individual species in that genus were listed in the same blocklist, the software would automatically remove the genus name from the list under the expectation that some but not all of its species lacked IRs. To avoid blocklisting taxa that do possess IRs, the search results are additionally compared against the internal survey results of airpg for cases where the presence of IRs has already been established.

### Structure of software

airpg was written in Python v.3.6 and consists of eight Python classes that coordinate the various aspects of automated data retrieval and data mining. Each of these classes employs the functionality of one or more third-party tools, which represent mandatory dependencies of airpg. Class *EntrezInteraction* coordinates the interactions with the interconnected databases of NCBI via the Entrez interface [33] and the download of sequence records. For a streamlined access to Entrez, the class employs the command-line tool *Entrez Direct* (often abbreviated as ‘*EDirect*’ [34]), which is used to conduct queries of NCBI Nucleotide and retrieve query results. Class *IROperations* coordinates the reading and writing of sequence records and the identification of IR annotations in these records. For reading and writing operations as well as the memory-efficient access to the annotation features, the class employs the Python package *Biopython* [35]. Class *TableIO* coordinates the data transfer between individual data frames on plastid genome accession numbers, metadata, IR presence and location, as well as duplicated and blocklisted taxa. For all internal operations of tabular data processing, the class employs the Python package *pandas* [36]. The classes *PubMedAnalyzer*, *PubMedRecord*, and *PubMedResult* coordinate the retrieval, parsing, processing, and storing of query responses to searches on NCBI PubMed. Class *PubMedAnalyzer* hereby utilizes the Python package *Entrezpy* [37] for the search and retrieval of PubMed article abstracts [38]. Class *ArticleMining* coordinates the data mining of article abstracts for keywords and plant taxon names. Specifically, the names of plant genera are identified through comparing all abstract words and keywords against the taxonomy database of NCBI for complete or partial matches. Class *ArticleMining* hereby utilizes the Python package *ete3* [39], which contains functionality to generate and scan the NCBI taxonomy database locally. Class *SelfBlasting* coordinates the process of confirming the length and location of the IRs of a plastid genome as specified through its sequence annotations with an automated self-comparison of the genome sequence (colloquially called ‘self-blasting’) using the command-line tool suite ‘*BLAST+*’ [40]. Specifically, the class identifies the true length and location of the IRs of a plastid genome record, if any, through a two-step process: first, a local database of the complete genome sequence is generated using command ‘*makeblastdb*’; second, the same genome sequence is compared against the database using command ‘*blastn*’.

While the eight classes of airpg coordinate the individual processes in identifying complete and correct IR annotations of a plastid genome, a full survey of plastid genomes on NCBI Nucleotide comprises a total of eleven operational steps. In step 1, any survey data of previous surveys as well as a list of known duplicate records and, optionally, a blocklist of names of plant species and genera without IRs are read in. In step 2, NCBI Nucleotide is queried based on an Entrez search string that defines the set of taxa to be surveyed. This query results in a primary list of record IDs (hereafter ‘UIDs’) which specifies the full set of plastid genomes to be probed for IR annotations. In step 3, the UIDs of previously processed records as well as the UIDs of known duplicate records are removed from the primary list, giving rise to a reduced secondary UID list. In step 4, the full record of each plastid genome specified in the secondary UID list is retrieved and parsed. Ten items of information are extracted from each record and written to file: the accession and the version number of the record, the species name, the sequence length, the date at which the record was first submitted to NCBI Nucleotide, the author names, title and journal of the reference publication, and the full taxonomic position of the species represented by the record. In step 5, each parsed record is mined for information on possible inclusion in the NCBI RefSeq database; positive hits are added to the list of known duplicates, and one of the duplicates is automatically removed from both the secondary UID list and the set of parsed records. In step 6, the complete information of each record is downloaded in GenBank flatfile format [41] unless a local copy of the record already exists. In step 7, the sequence annotations of each record are analyzed to identify any annotations that explicitly or implicitly specify IRs using the set of identification criteria implemented in class *IROperations*; the presence, length, and location of any IR so identified are recorded. In step 8, the complete sequence of each genome record as well as the sequences of each IR, if present, are written to file in FASTA format. In step 9, PubMed is queried for articles that contain information on genera that naturally lack IRs; positive hits are parsed, and the taxon names added to the blocklist. In step 10, taxa that have been found through the PubMed article search, but for which class *IROperations* has successfully identified two IRs, are removed from the blocklist as false positives of the PubMed article search. In step 11, the IR annotations of each genome record are confirmed by comparing the complete genome sequence against itself using *blastn*; the length and location of the IRs so identified are written to file, with the original annotations and their BLAST-based re-evaluation listed side-by-side for easy comparison.

### Operation of software

airpg is operated via four wrapper scripts that are co-supplied with the package. Each script produces a detailed log to inform the user of its current operations. The first script, named airpg_identify.py, automates the operation of steps 1 through 3 of a full survey and, thus, coordinates the query of NCBI Nucleotide; it also accommodates the information of previous survey results, a list of duplicate records, and a potential taxon blocklist (Additional file 1: Figure S2). If the output from a previous run is supplied to airpg_identify.py as input, the script will append only new records to the output; the list of duplicate records is also extended, if applicable. Script airpg_identify.py accepts one mandatory and three optional command-line parameters as input. Parameter -o/--outfn is mandatory and requires a valid file path to the output table. The list of duplicate records is also defined by this parameter, as any information on duplicate accessions is loaded from, and saved to, a file with the same name extended by the word ‘.duplicates’. Parameter -q/--query is optional and accepts a user-supplied Entrez search string. By default, this parameter is set to a search of all complete and verified plastid genomes of flowering plants with a genome length between 50 kb and 250 kb. Parameter -b/--blocklist is optional and accepts a valid file path to the list of blocklisted taxa. This list must be a plain text file with one species or genus name per line. If the parameter is not provided, taxa will not be removed from the query results. Parameter -u/--update_only is an optional flag that compels the script to process only records published since the last run of the script. The primary output of script airpg_identify.py is a tab-delimited table of plastid genome records that match the criteria of the query search string. This table represents the secondary UID list and contains eleven data columns (UID; accession number; accession version; species name; sequence length; date of submission to NCBI Nucleotide; author names; title of reference publication; journal of reference publication; a note indicating duplicate records (if any); the full taxonomic position of the species) and as many rows (plus one for the column names) as unique, non-blocklisted plastid genome records were identified by the query. The secondary output of the script is a tab-delimited table of duplicate records, which connects the accession number of each record on NCBI Nucleotide to the corresponding UID and accession number on the RefSeq database, if existent. Only the RefSeq record is retained, whereas the corresponding NCBI Nucleotide record is listed as duplicate and removed from the primary output at run time.

The second script, named airpg_analyze.py, automates the operation of steps 4 through 10 of a full survey and, thus, coordinates the retrieval and parsing of the full record of each plastid genome specified in the secondary UID list, the parsing of information on duplication in NCBI RefSeq, the data mining of the annotation features for the length and location of the IR regions, the query of NCBI PubMed for taxa that naturally lack IRs, and the nucleotide sequence extraction of the IRs and the complete genome (Additional file 1: Figure S3). If the output from a previous run is supplied as input, the script will append only new records to the output. Script airpg_analyze.py accepts three mandatory and three optional command-line parameters as input. Parameter -i/--infn is mandatory and requires a valid file path to the output table generated by script airpg_identify.py. Parameter -o/--outfn is mandatory and requires a valid file path to its own output table to which the annotation-based information on IR presence, length, and location is written. Parameter -m/--mail is mandatory and requires a valid email address, which is needed to execute an Entrez query on NCBI PubMed. The parameters -r/--recordsdir and -d/--datadir are optional and accept valid directory paths to which the retrieved plastid genomes and their individual IRs (if any) are saved as compressed flat- and sequence files, respectively. If no directory parameter is provided by the user, new sub-directories (with the folder names ‘records’ and ‘data’) are created in the current working directory. Parameter -b/--blocklist is optional and accepts a valid file path to the list of blocklisted taxa. The output of script airpg_analyze.py is a tab-delimited table containing annotation-based information on the presence, length, and location of the IRs of each plastid genome record. It contains nine data columns (accession number; presence value of ${\text{IR}}_{B}$; start position of ${\text{IR}}_{B}$; end position of ${\text{IR}}_{B}$; length of ${\text{IR}}_{B}$; presence value of ${\text{IR}}_{A}$; start position of ${\text{IR}}_{A}$; end position of ${\text{IR}}_{A}$; length of ${\text{IR}}_{A}$) and as many rows (plus one for the column names) as plastid genome records were listed in the input to this script.

The third script, named airpg_update_blocklist.py, generates a basic blocklist of taxa that naturally lack IRs in their plastid genomes or appends an existing blocklist with additional taxa through an automated query of NCBI PubMed. The script accepts one mandatory and two optional command-line parameters as input. Parameter -f/--file_blocklist is mandatory and requires a valid file path to an empty or a previously generated taxon blocklist. Parameters -q/--query and -m/--mail are optional and accept an Entrez search string and a valid email address, respectively. The search string is the basis for the query of NCBI PubMed and aims to identify taxa beyond the IRL clade of the Fabaceae that naturally lack IRs in their plastid genomes. By default, this search string is set to retrieve the names of all plant genera in PubMed article abstracts whose abstract titles contain each of the keywords ‘inverted’, ‘repeat’, and ‘loss’.

The fourth script, named airpg_confirm.py, automates the operation of step 11 of a full survey and, thus, coordinates the confirmation of the IR annotations of each genome record through sequence self-comparisons using *BLAST+*. The length and location of any IRs identified through self-blasting the genome sequence are saved side-by-side with the original IR annotation information as an expanded version of the table generated by script airpg_analyze.py. Script airpg_confirm.py accepts three mandatory and two optional command-line parameters as input. Parameter -i/--infn is mandatory and requires a valid file path to the table generated by script airpg_analyze.py, which represents one of the inputs to this script. Parameter -o/--outfn is mandatory and requires a valid file path to which an expanded version of the input table is written as output. Parameter -d/--datadir is mandatory and requires a valid path to the directory hosting the record-specific sub-directories that contain the complete genome sequences in FASTA format. Parameters -n/--minlength and -x/--maxlength are optional and accept the minimum and maximum length, respectively, of any repeat region that is to be identified as plastid IR; by default, these parameters are set to 10 kb and 50 kb, respectively, to cover the plastid IR length of virtually all flowering plants [1, 3]. The output of script airpg_confirm.py is a tab-delimited table that contains eight additional data columns compared to the input table and which specify the presence, length, and location of the IRs as determined through self-blasting the genome sequences.

In each of the four scripts of airpg, the full set of available command-line parameters, their default values, and a short explanation of each parameter can be displayed by invoking parameter -h/--help. airpg is accessible via the Python package index under http://pypi.python.org/pypi/airpg and can be installed including all Python dependencies using the command pip install airpg. The software has been successfully tested on Arch Linux 5.9.14, Debian 10.6, and Ubuntu 20.10.

### Evaluation of software on empirical data

To illustrate the functionality of airpg on large-scale empirical data, a full survey of the complete- and correctness of IR annotations among plastid genomes of flowering plants was conducted. The survey targeted all complete and verified plastid genomes of flowering plants that were submitted to NCBI Nucleotide during the 20-year period between the beginning of January 2000 and the end of December 2020 and that exhibited a sequence length between 50 kb and 250 kb, which represents the empirical length range of plastid genomes of photosynthetically active flowering plants [2, 14]. The completeness of the IR annotations of each genome record was determined by the presence of sequence annotations for both IRs and evaluated separately from the assessment of length equality between these annotations. The correctness of the IR annotations of each genome record was determined by confirming these annotations regarding presence, equal length, and location through self-blasting the genome sequence. The aims of our survey were to assess (i) the frequency of IR annotations among all genome records under study regarding completeness, equal length, and correctness, and (ii) potential effects between the frequency of complete IR annotations and any of four descriptive factors associated with the genome records. Specifically, we assessed the presence of complete and equal-length IR annotations in comparison to (a) release year, (b) publication status, (c) record version number, and (d) taxonomic position regarding plant family. Statistical effects of release year, publication status, and record version number were assessed in R v.4.0.3 [42] using generalized linear models (GLMs). The presence/absence of complete and equal-length IR annotations was employed as binary response variable, release year, publication status, and record version number as predictor variables. The significance of the effect of the predictor variables on the response variable was evaluated at $p<0.05$. The frequency of complete and equal-length IR annotations per plant family was listed for different families and compared against the absolute number of records per family as well as the overall number of records for all flowering plants. A standard blocklist was employed to exclude plastid genomes that naturally lack IRs from the survey. For reference and reproducibility, all files generated during the survey were deposited to Zenodo at https://zenodo.org/record/4772615. Except for the given email address, which was specified correctly, the following commands were invoked in a terminal/shell to conduct the survey:
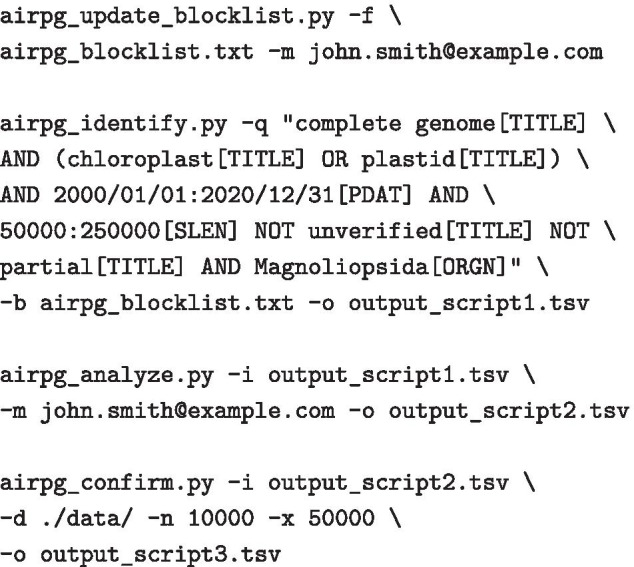


## Results

### Complete- and correctness of IR annotations

**Fig. 1:**
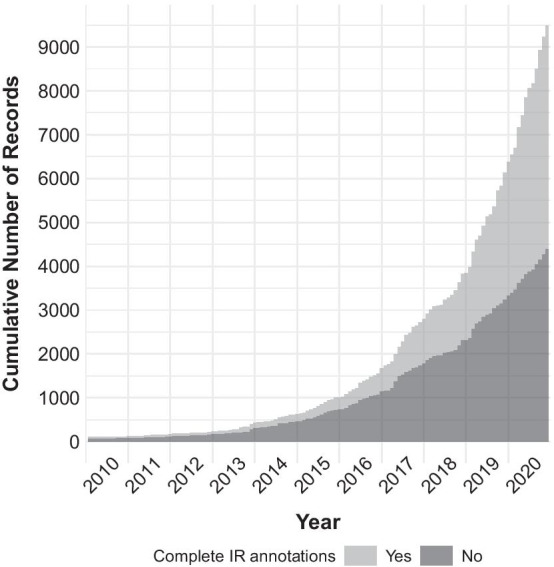
Accumulation of plastid genomes of flowering plants with and without complete IR annotations. Displayed is the accumulation of plastid genomes between January 2010 and December 2020, with different gray shades highlighting the presence (light gray) or absence (dark gray) of complete IR annotations

**Fig. 2:**
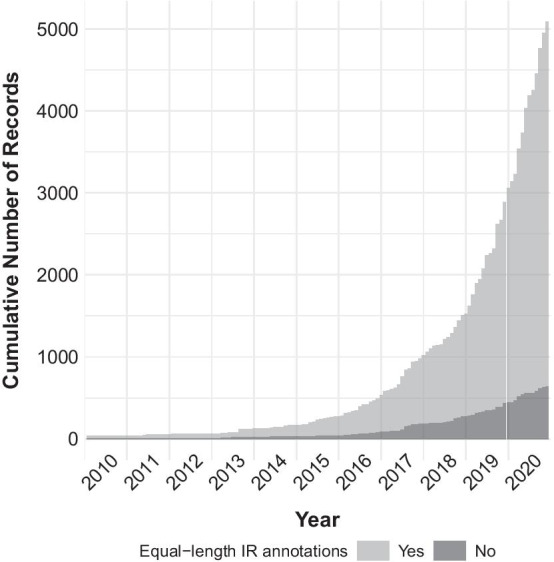
Accumulation of plastid genomes of flowering plants with and without equal-length IR annotations. Displayed is the accumulation of plastid genomes with complete IR annotations between January 2010 and December 2020, with different gray shades highlighting the presence (light gray) or absence (dark gray) of equal-length IR annotations per genome

The number of unique and complete plastid genomes of flowering plants on NCBI Nucleotide has increased dramatically over the past decade but less than half of these genomes contain complete IR annotations. We employed the output of script airpg_identify.py in conjunction with that of script airpg_analyze.py to visualize the accumulation of plastid genomes with and without complete IR annotations for the second half (i.e., January 2010 to December 2020) of the 20-year period under study (Fig. 1). During this second half, the number of plastid genome records increased from 99 to 9483, which represents an almost 100-fold increase. During 2019 alone, the number of records increased by 60% from 3822 to 6132 records. Moreover, by the end of December 2020 the number of unique and complete plastid genome records on NCBI Nucleotide was almost twice as large as the equivalent number on NCBI RefSeq (https://ftp.ncbi.nlm.nih.gov/refseq/release/plastid/; release of 2020-11-07) and covered a total of 294 different families of flowering plants. However, a considerable number of these genome records exhibited incomplete IR annotations: 65% of all records contained neither explicit nor implicit IR annotations in January 2010, and 46% continued to do so in December 2020 (Fig. 1). Similarly, a non-negligible number of genome records with complete IR annotations suggested unequal IR lengths: 9% of all records with complete IR annotations implied length differences between ${\text{IR}}_{A}$ and ${\text{IR}}_{B}$ in 2014, and a similar percentage of all records implied IR length inequality in 2020 (Fig. 2).

**Fig. 3:**
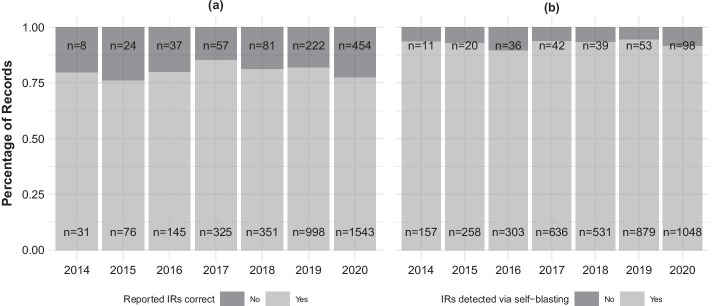
Frequencies of plastid genomes of flowering plants with and without correct IR annotations. Displayed are yearly frequencies of genome records between 2014 and 2020. **a** The frequency of records with complete and equal-length IR annotations in which these annotations are correct (light gray) or incorrect (dark gray) given the genome sequences. **b** The frequency of genome records without complete IR annotations in which IRs do (light gray) or do not (dark gray) exist in the genome sequences

Our analyses also indicated that the reported IR annotations of a considerable number of plastid genome records were incorrect, even though these annotations were complete and implied equal IR lengths. Specifically, we employed the output of script airpg_identify.py in conjunction with that of script airpg_confirm.py to visualize the frequency of correct and incorrect IR annotations among plastid genomes with and without complete annotations of these repeats. We found that between 15% and 24% of all annual plastid genomes submitted to NCBI Nucleotide between 2014 and 2020 reported complete and equal-length, yet incorrect IR annotations (Fig. 3a). Moreover, we found that between 89% and 94% of all annual plastid genomes without complete IR annotations that were submitted to NCBI Nucleotide during the same time period did exhibit ${\text{IR}}_{A}$ and ${\text{IR}}_{B}$ in their sequences; conversely, only between 6% and 11% of all annual plastid genomes without complete IR annotations genuinely lacked IRs (Fig. 3b).

### IR annotation completeness and metadata

**Fig. 4:**
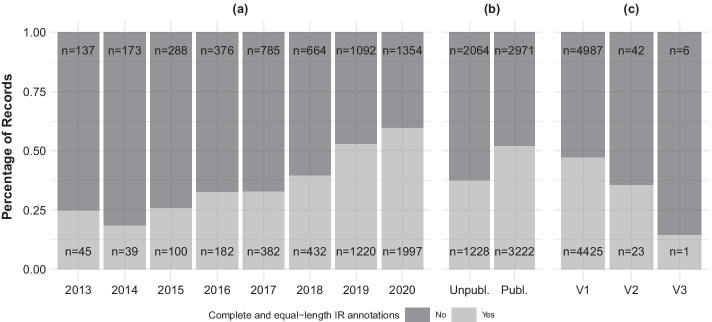
Frequencies of plastid genomes of flowering plants with and without complete and equal-length IR annotations in relation to **a** release year, **b** publication status, and **c** record version number. Different gray shades highlight the presence (light gray) or absence (dark gray) of complete and equal-length IR annotations. In (**a**), annual frequencies between 2013 and 2020 are given

Two factors associated with the plastid genome records under study were found to have significant effects on the frequency of complete and equal-length IR annotations among these records. The results of our GLM tests indicated that release year and publication status of the records were significantly associated with the presence of complete and equal-length IR annotations (Table 1). By contrast, we were unable to reject the null hypothesis that record version number was unassociated with the presence of such IR annotations. Factor-specific analyses indicated that the annual frequency of complete and equal-length IR annotations had steadily increased per release year since 2014, and that such IR annotations were found in less than half of all annual submissions before and including 2018, but in more than half of all annual submissions after 2018 (Fig. 4a). Our factor-specific analyses also indicated that published plastid genome records contained complete and equal-length IR annotations more often than unpublished records: 52% of published records but only 37% of unpublished records contained such IR annotations (Fig. 4b). The factor-specific analyses further indicated that an increase in version number (i.e., a higher number of record revisions) was negatively correlated with the frequency of complete and equal-length IR annotations (Fig. 4c); however, only 72 (i.e., less than 1‰) of the plastid genome records under study have been revised on NCBI Nucleotide, rendering the comparison highly unbalanced.

**Table 1:** Statistical effects and confidence intervals of the frequency of complete and equal-length IR annotations with regard to release year, publication status, and record version number

	Effect values				Conf. intervals	
	estim.	std. err.	stat.	*p* value	2.5%	97.5%
V.1	0.06	0.28	0.22	8.24e–1	−0.501	0.606
V.2	−1.1	1.15	−0.96	3.39e–1	−4.105	0.890
V.3	15.0	119.0	0.13	9.00e–1	−9.324	NA
Year	0.25	0.012	21.3	1.06e–100*	0.226	0.271
Publ.	0.66	0.045	14.5	1.01e–47*	0.569	0.746

*p* values indicating significance are indicated by an asterisk, instances when models cannot be fitted to the data by ‘NA’

v., sequence version; estim., estimate; stat., statistic; std.err., standard error

Our evaluation of the frequency of complete and equal-length IR annotations across plant families indicated strong frequency differences: among the ten families of flowering plants with the highest number of plastid genome records on NCBI Nucleotide, the percentage of records with complete and equal-length IR annotations ranged between 85.7% in the Melastomataceae and 17.9% in the Solanaceae (Table 2). The Poaceae represent the flowering plant family with the highest number of unique plastid genome records on NCBI Nucleotide and account for 13.3% of all flowering plant plastid genome records submitted there until the end of 2020, yet less than one-third of their records exhibit complete and equal-length IRs annotations.

**Table 2:** Absolute and relative numbers of plastid genome records per family of flowering plants and the share of records within each family that exhibit complete and equal-length IR annotations. Only the top ten families with regard to the absolute record number are displayed and are sorted by that number

Plant family	Abs. num.	Rel. num. (%)	Share (%)
Poaceae	1263	13.32	31.27
Asteraceae	494	5.32	24.29
Orchidaceae	421	4.44	60.10
Solanaceae	418	4.41	17.94
Brassicaceae	413	4.35	71.19
Rosaceae	353	3.72	64.87
Fabaceae	336	3.54	53.87
Ranunculaceae	189	1.99	58.20
Apiaceae	184	1.94	58.15
Melastomataceae	182	1.92	85.71

abs., absolute; num., number; rel., relative

## Discussion

Our survey of the complete- and correctness of IR annotations among plastid genome records via airpg highlighted the importance of software tools that assess the quality of sequence annotations in an automated fashion. The results of our survey allowed the identification of significant effects by release year and publication status on the completeness of IR annotations among archived plastid genomes. Specifically, we found an increased frequency of complete and equal-length IR annotations among plastid genomes that were released as part of a scientific publication as opposed to genomes that were merely uploaded to the database. This increase in frequency may be the result of a higher diligence in sequence annotation of genome records that face peer-review and scientific scrutiny. Similarly, we found an increase in complete and equal-length IR annotations among plastid genomes that have been released since 2014, as opposed to records released before that time. This increase in frequency may be indicative of a growing awareness among researchers of the importance of correctly annotated genome records [24, 25], as well as a rising availability of software tools that automate the annotation process for organellar genomes [15, 43]. By contrast, the decreased frequency of complete and equal-length IR annotations identified among plastid genome records that have undergone revisions of either their sequence or sequence annotations as opposed to unrevised records likely represents a statistical artifact, as less than 1‰ of all records under study represent revised genome records. The logical assumption that annotation quality improves with a higher number of record revisions may, thus, hold true, even if the current numbers do not corroborate it. Taken together, these findings highlight the need for the continued development of software tools that can automatically assess and process the sequence annotations of organellar genomes, as ever larger amounts of genome sequence data are generated in scientific investigations and require quality assessment.

## Conclusions

The number of plastid genomes deposited to NCBI Nucleotide has increased dramatically in recent years, and thousands of additional plastid genomes will likely be submitted over the next decade. The IRs of a plastid genome represent a characteristic genome feature, yet more than half of all plastid genome records on NCBI Nucleotide do not exhibit complete annotations for them. The Python package airpg enables researchers to automatically access and survey the IR annotations of plastid genomes archived on NCBI Nucleotide and, thus, to conduct important evaluations of annotation quality and the factors influencing that quality. In an empirical survey, we found that release year, publication status, and possibly taxonomic position affect the presence of complete and equal-length IR annotations in plastid genome records. The causes behind these and similar effects should be further investigated, and airpg provides a helpful tool for such analyses.

## Availability and requirements

- *Project name* airpg
- *Project home page*http://pypi.python.org/pypi/airpg, https://github.com/michaelgruenstaeudl/airpg
- *Operating systems* Linux
- *Programming language* Python ($>=$ 3.6)
- *Other requirements* Command-line tools *Entrez Direct* and *BLAST+*; Python libraries *biopython*>=1.72, *entrezpy*, *ete3*, and *pandas*; a reasonably fast internet connection
- *License* GNU General Public License
- *Any restrictions to use by non-academics* none

## Supplementary information

**Additional file 1**. Workflow of three different processes in airpg: the identification of inverted repeats (Fig. S1), the operation of script *airpg_identify.py* (Fig. S2), and the operation of script *airpg_analyze.py* (Fig. S3).

**Additional file 2**. Bash code for, and results of, a comparison of the number of plastid genome records of flowering plants stored on NCBI Genome versus those stored on NCBI Nucleotide.

## Data Availability

airpg is available under the GNU General Public License via the Python package index at http://pypi.python.org/pypi/airpg. The data sets supporting the results of this investigation are available on Zenodo at https://zenodo.org/record/4772615. An interactive version of the package and the command-line code of four different example surveys are available on CodeOcean under https://codeocean.com/capsule/6723913/tree/v1.

## Abbreviations

bp: Base pairs
GLM: Generalized linear model
IR: Inverted repeat
IRL: Inverted repeat-lacking
kb: Kilo base pairs
LSC: Large single copy
NCBI: National Center for Biotechnology Information
SC: Single copy
SSC: Small single copy
