# Supplementary material for: airpg: automatically accessing the inverted repeats of archived plastid genomes
Source: BMC Bioinformatics. 2021 Aug 21;22:413. doi: 10.1186/s12859-021-04309-y (PMC8379869; doi:10.1186/s12859-021-04309-y)

Figure S 1: Workflow of the identification of inverted repeats in `airpg`.

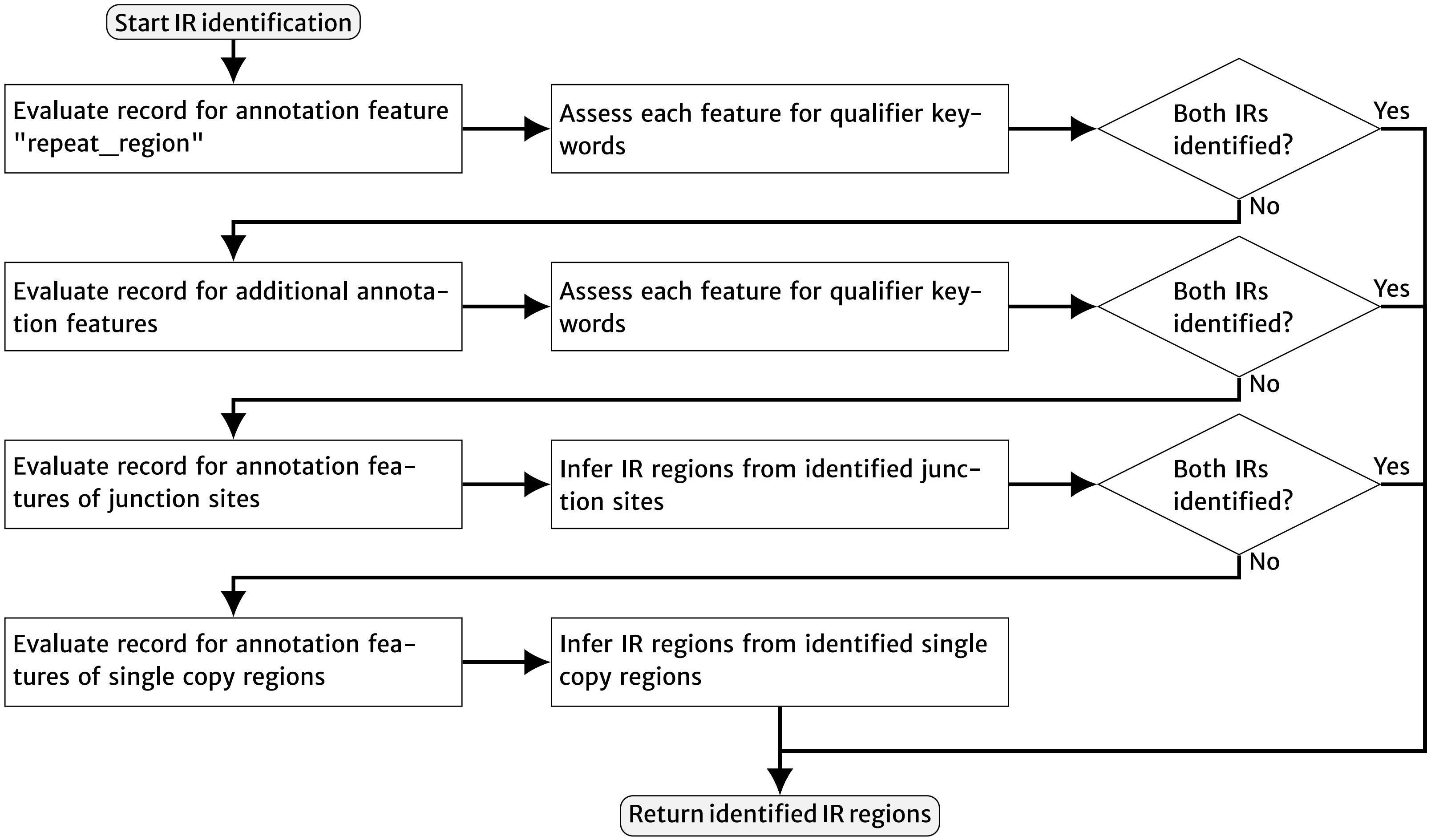

Figure S 2: Workflow of script `airpg_identify.py`. Input and output are indicated in gray and, if potentially identical, appended with the keywords 'input' and 'output' in parentheses.

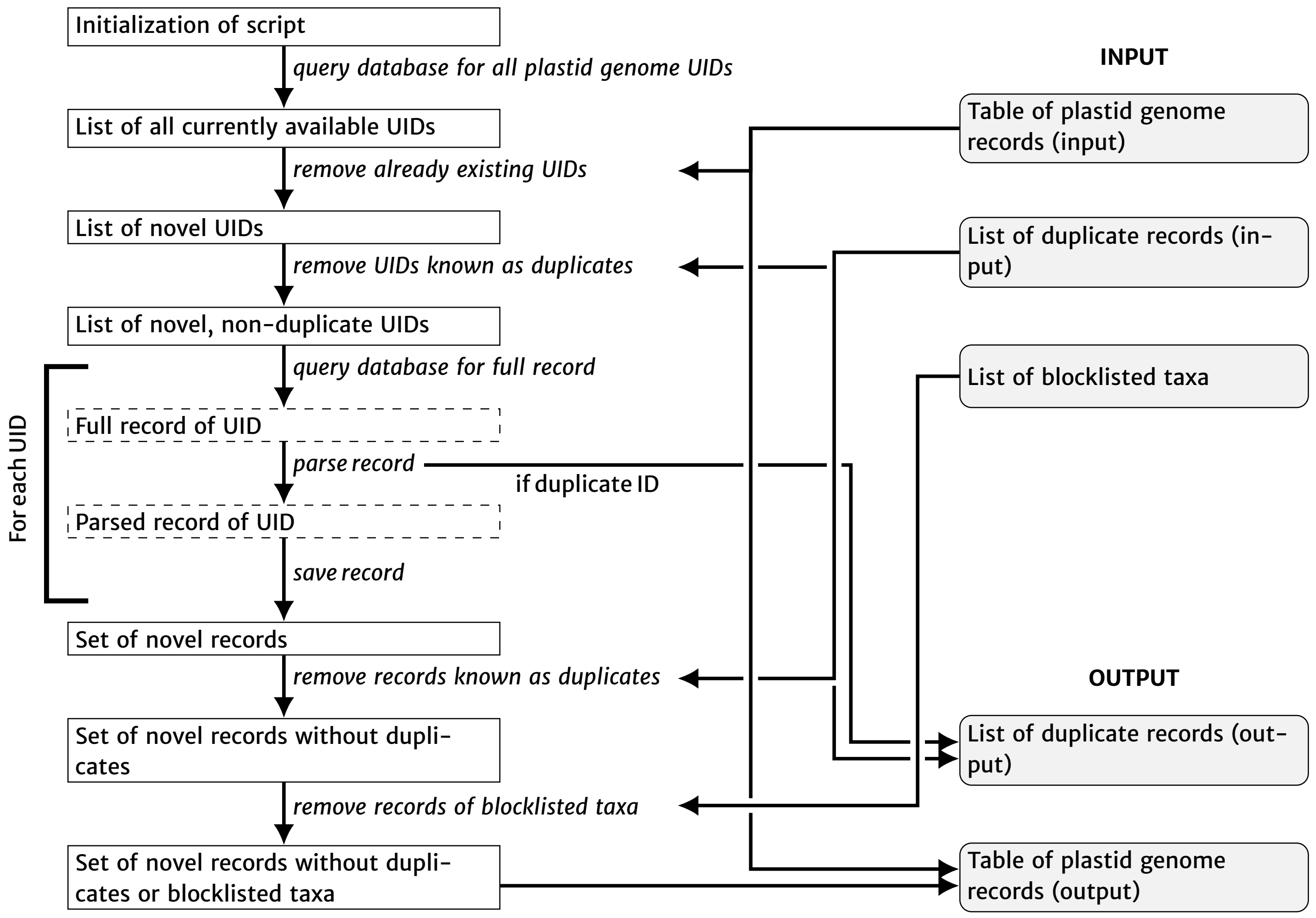

Figure S 3: Workflow of script airpg_analyze.py. Input and output are indicated in gray and, if potentially identical, appended with the keywords 'input' and 'output' in parentheses.

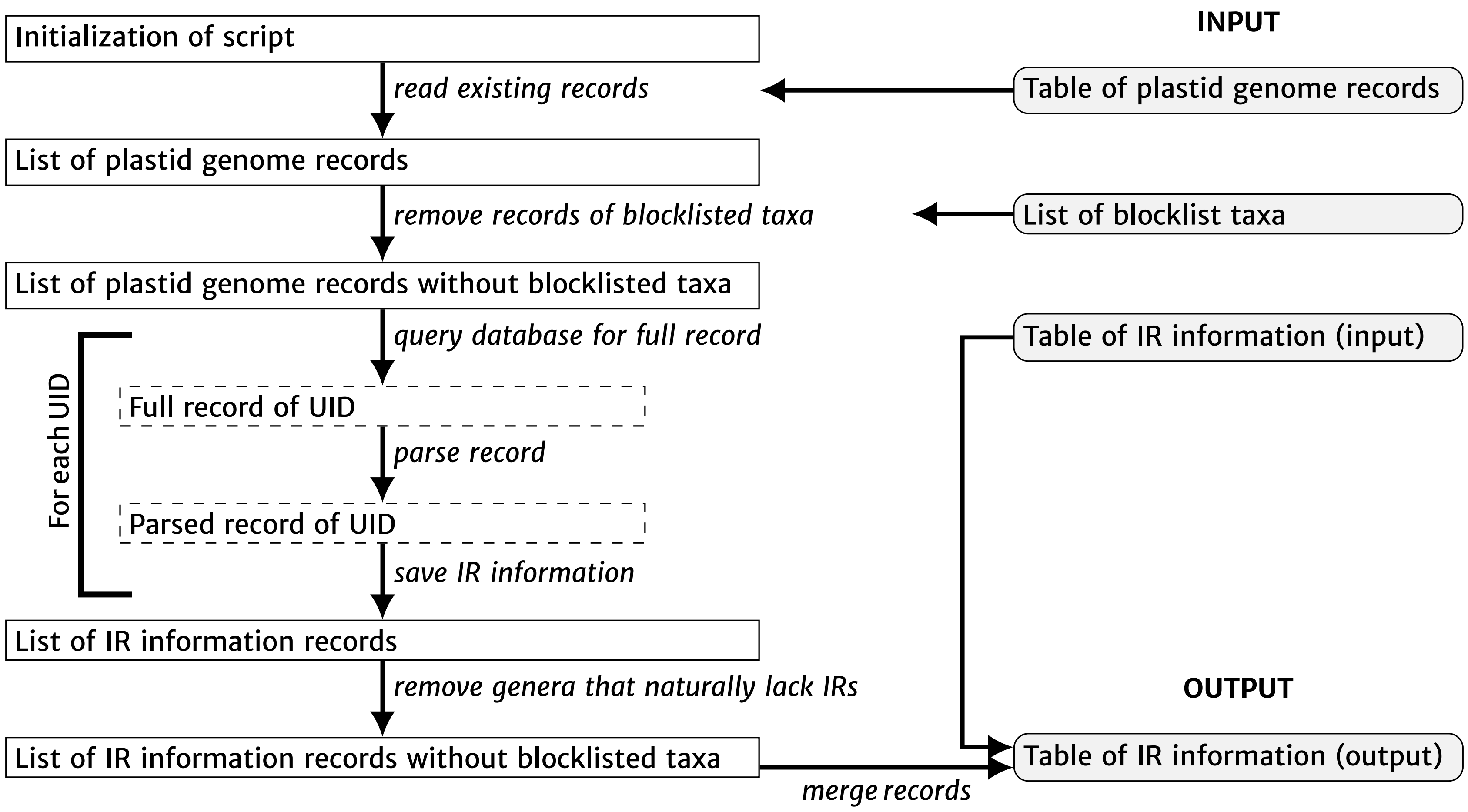

Supplement: Supplementary file 1 — Additional file 1. Workflow of three different processes in airpg: the identification of inverted repeats (Fig. S1), the operation of script airpg_identify.py (Fig. S2), and the operation of script airpg_analyze.py (Fig. S3). [file 12859_2021_4309_MOESM1_ESM.pdf]
