# Supplementary material for: airpg: automatically accessing the inverted repeats of archived plastid genomes
Source: BMC Bioinformatics. 2021 Aug 21;22:413. doi: 10.1186/s12859-021-04309-y (PMC8379869; doi:10.1186/s12859-021-04309-y)
Supplement: Supplementary file 2 — Additional file 2. Bash code for, and results of, a comparison of the number of plastid genome records of flowering plants stored on NCBI Genome versus those stored on NCBI Nucleotide. [file 12859_2021_4309_MOESM2_ESM.pdf]

Bash code for, and results of, a comparison of the number of plastid genomes of flowering plants stored on NCBI Genome versus those stored on NCBI Nucleotide. The yellow code block displays the Bash code employed to retrieve all plastid genome records of flowering plants stored on NCBI Genome under the same sequence length constraints as employed in the search of NCBI Nucleotide. The blue code block presents the number of plastid genome records retrieved from NCBI Genome. The red code block displays the Bash code employed to infer the intersection and the two complement sets of plastid genome records of flowering plants stored on NCBI Genome and NCBI Nucleotide.

```bash

#!/bin/bash

# AUTHOR="Michael Gruenstaeudl, PhD"
# CONTACT="m.gruenstaeudl@fu-berlin.de"
# VERSION="2021.05.06.1800"

###################################
## SPECIFY INPUT AND OUTPUT FILE ##
###################################
## On 06-May-2021, the input list is downloaded from
## https://www.ncbi.nlm.nih.gov/genome/browse#!/organelles/
## upon making the following selections:
## Subgroup: Land Plants
## Type: chloroplast, plastid
INF=plastid_chloroplast_LandPlants__2021_05_06_1800.csv
HDL=${INF%.csv*}__HANDLE.csv
OTF=plastid_chloroplast_LandPlants__2021_05_06_1800__mySelection.csv

###################################
## ADD LENGTH AND TAXONOMIC INFO ##
###################################
# Set up fresh outfile
echo -n "" > ${HDL%.csv*}_1.csv
# Loop through lines of infile
while IFS="" read -r line || [[ -n "$line" ]]
do
    echo -n "$line" >> ${HDL%.csv*}_1.csv
    # Parse out NC_number
    NCNUM=$(echo "$line" | awk -F"," '{print $9}' | \
      awk -F"/" '{print $1}' | tr -d '"' | sed 's/\..//')
    if [[ $NCNUM == NC_* ]]
```

```
    then
        echo "Processing $NCNUM ..."
        # Get GenBank flatfile for NC_number
        RECORD=$(esearch -db nucleotide -query "$NCNUM" </dev/null | \
          efetch -format gb)
        # Parse out and save sequence length
        echo -n "$RECORD" | grep "^LOCUS" | awk '{print ","$3}' | \
          tr -d "\n" >> ${HDL%.csv*}_1.csv
        # Parse out taxonomic information
        TAXINFO=$(echo "$RECORD" | grep -A4 "^  ORGANISM" | sed 1d | \
          tr -s " " | tr -d "\n" | awk '{$1=$1}1' | \
          awk '{print "\""$0"\""}')
        echo -n ",$TAXINFO" >> ${HDL%.csv*}_1.csv
        # Test if keyword "Magnoliophyta" in taxonomic information
        if [[ $TAXINFO == *"Magnoliophyta"* ]] || \
           [[ $TAXINFO == *"Magnoliopsida"* ]]
        then
            echo ",Magnoliophyta" >> ${HDL%.csv*}_1.csv
        else
            echo "," >> ${HDL%.csv*}_1.csv
        fi
    else
        echo ",n.a.,n.a.,n.a." >> ${HDL%.csv*}_1.csv
    fi
done < $INF

################################
## REMOVE ALL NON-ANGIOSPERMS ##
################################
echo "Enforcing taxonomic constraints ..."
# Loop through lines of infile
while IFS="" read -r line || [[ -n "$line" ]]
do
    KEYFIELD=$(echo "$line" | awk -F"," '{print $21}')
    if [[ $KEYFIELD == "Magnoliophyta" ]]
    then
        echo "$line" >> ${HDL%.csv*}_2.csv
    else
        echo "$line" >> ${HDL%.csv*}_2_removed.csv
    fi
done < ${HDL%.csv*}_1.csv

```

```
########################################
## KEEP ONLY PLASTOMES IN LENGTH RANGE ##
########################################
echo "Enforcing genome length constraints ..."
# Loop through lines of infile
while IFS="" read -r line || [[ -n "$line" ]]
do
    KEYFIELD=$(echo "$line" | awk -F"," '{print $19}')
    if [[ $KEYFIELD -ge 49999 ]] && [[ $KEYFIELD -le 250001 ]]
    then
        echo "$line" >> ${HDL%.csv*}_3.csv
    else
        echo "$line" >> ${HDL%.csv*}_3_removed.csv
    fi
done < ${HDL%.csv*}_2.csv

#####################################
## KEEP ONLY PLASTOMES YOUNGER THAN ##
#####################################
echo "Enforcing publication date constraints ..."
# Loop through lines of infile
while IFS="" read -r line || [[ -n "$line" ]]
do
    KEYFIELD=$(echo "$line" | awk -F"," '{print $11}' | \
      sed 's/T00:00:00Z//g' | tr -d '"')
    cutoff=$(date -d"2020-12-31" "+%Y-%m-%d")
    testdate=$(date -d"$KEYFIELD" "+%Y-%m-%d")
    if [[ $testdate < $cutoff ]]
    then
        echo "$line" >> ${HDL%.csv*}_4.csv
    else
        echo "$line" >> ${HDL%.csv*}_4_removed.csv
    fi
done < ${HDL%.csv*}_3.csv

#############
## SUMMARY ##
#############
echo -ne "\nNumber of sequence records in input file: "
if [[ -f $INF ]]; then
    cat $INF | grep -v "^#Organism" | wc -l
else
```

```
    echo 0
fi
echo -ne "\nNumber of records removed for not being angiosperm: "
if [[ -f ${HDL%.csv*}_2_removed.csv ]]; then
    cat ${HDL%.csv*}_2_removed.csv | wc -l
else
    echo 0
fi
echo -ne "\nNumber of records removed for not being in length range: "
if [[ -f ${HDL%.csv*}_3_removed.csv ]]; then
    cat ${HDL%.csv*}_3_removed.csv | wc -l
else
    echo 0
fi
echo -ne "\nNumber of records removed for not being in date range: "
if [[ -f ${HDL%.csv*}_4_removed.csv ]]; then
    cat ${HDL%.csv*}_4_removed.csv | wc -l
else
    echo 0
fi
echo -ne "\nNumber of records that fulfill all criteria: "
if [[ -f ${HDL%.csv*}_4.csv ]]; then
    cat ${HDL%.csv*}_4.csv | wc -l
else
    echo 0
fi

#################
## FILE HYGIENE ##
#################
cp ${HDL%.csv*}_4.csv $OTF
tar -czf ${OTF%.csv*}.tar.gz ${HDL%.csv*}*
rm *_HANDLE*
```

Results:

```
Number of sequence records in input file: 6080
Number of records removed for not being angiosperm: 625
Number of records removed for not being in length range: 19
Number of records removed for not being in date range: 511
Number of records that fulfill all criteria: 4926
```

Comparison of files:

```
comm -23 \
    <(cat output_script2.tsv.col1.sorted) \
    <(cat plastid_chloroplast_LandPlants__2021_05_06_1800__mySelection.csv.col9.sorted) > accessions_unique_to__output_script2.txt

echo -n "Number of accessions unique to file 'output_script2.tsv': "
comm -23 \
    <(cat output_script2.tsv.col1.sorted) \
    <(cat plastid_chloroplast_LandPlants__2021_05_06_1800__mySelection.csv.col9.sorted) | wc -l
# Number of accessions unique to file 'output_script2.tsv': 4662

##########

comm -13 \
    <(cat output_script2.tsv.col1.sorted) \
    <(cat plastid_chloroplast_LandPlants__2021_05_06_1800__mySelection.csv.col9.sorted) >
    accessions_unique_to__plastid_chloroplast_LandPlants__2021_05_06_1800__mySelection.csv

echo -n "Number of accessions unique to file 'plastid_chloroplast_LandPlants__2021_05_06_1800__mySelection.csv': "
comm -13 \
    <(cat output_script2.tsv.col1.sorted) \
    <(cat plastid_chloroplast_LandPlants__2021_05_06_1800__mySelection.csv.col9.sorted) | wc -l
# Number of accessions unique to file 'plastid_chloroplast_LandPlants__2021_05_06_1800__mySelection.csv': 105

##########

comm -12 \
    <(cat output_script2.tsv.col1.sorted) \
    <(cat plastid_chloroplast_LandPlants__2021_05_06_1800__mySelection.csv.col9.sorted) > accessions_shared_by_both_files.csv

echo -n "Number of accessions shared by both files: "
comm -12 \
    <(cat output_script2.tsv.col1.sorted) \
    <(cat plastid_chloroplast_LandPlants__2021_05_06_1800__mySelection.csv.col9.sorted) | wc -l
# Number of accessions shared by both files: 4820
```
